# Clinical characteristics of Korean asthmatic patients who showed negative skin prick tests, but positive specific IgE tests for fungus

**DOI:** 10.1186/1939-4551-8-S1-A260

**Published:** 2015-04-08

**Authors:** Byoung Whui Choi

**Affiliations:** 1 Chung-Ang University Hospital, South Korea

## Background

Sensitivity to allergens has been recognized as a risk factor for the development of asthma. There was few study about clinical significances of positive specific IgE Tests for fungus in patients with negative skin prick tests for fungus.

## Objective

We aimed to investigate clinical characteristics of asthmatics who showed negative skin prick tests, but positive specific IgE tests for fungus. The clinical difference between serum fungus-specific IgE-positive and –negative asthmatics who showed negative skin prick tests was also examined.

## Method

A total of 38 patients who showed negative skin prick tests after undergoing 50 common allergens including D. farinae, D. pteronyssiuns and 5 fungi (*Penicillium notatum*, *Cladosporium*, *Aspergillus fumigatus*, *and Alternaria*,*Fusarium spp.*) with positive methacholine provocation test provocation test and chronic lower respiratory symptoms were included in this study.

## Results

In 16 patients (42.1%) with negative serum specific IgE tests for both fungi and HDM, total IgE ( 76.1 + 64.7 KU/L) was significantly lower than in 14(36.8%) patients with positive serum specific IgE tests for fungi (730.1 + 948.41 KU/L) and in 16 patients (42.1%). with positive serum specific IgE tests for HDM (783.5 + 854.1 KU/L), respectively. In patients with negative serum specific IgE tests for both fungi and HDM, FEV1 % predicted (95.9 + 19.2 %) was significantly higher than in patients with positive serum specific IgE tests for fungi (75.5 + 24.2 % )and in patients with positive serum specific IgE tests for HDM (76.7 + 27.3 % ), respectively. In patients with negative serum specific IgE tests for both fungi and HDM, PC20 (2.61 + 1.32 mg/ml) was significantly higher than in patients with positive serum specific IgE tests for fungi (2.23 + 2.05 mg/ml) and in patients with positive serum specific IgE tests for HDM (1.34 + 1.12 mg/ml), respectively.

## Conclusion

In asthmatic patients who showed negative skin prick tests, but high serum IgE, serum specific igE test for both fungi and HDM might be needed for detection of HDM.

